# DMEK graft preparation techniques - liquid bubble technique compared to Melles technique

**DOI:** 10.1007/s00417-026-07238-9

**Published:** 2026-04-14

**Authors:** Jan-Philipp Bodenbender, Annekatrin Rickmann, Johanna Wude, Jens Martin Rohrbach, Karl Ulrich Bartz-Schmidt, Sebastian Thaler, Tarek Bayyoud

**Affiliations:** 1 Centre for Ophthalmology, University Eye Hospital, University of Tübingen, Elfriede-Aulhorn-Str. 7, 72076 Tübingen, Germany; 2 Eye Clinic Sulzbach, Knappschaft Hospital Saar, Sulzbach/Saar, Germany

**Keywords:** Cornea, DMEK, Fuchs endothelial corneal dystrophy, Bullous keratopathy, Liquid bubble technique, Melles technique

## Abstract

**Purpose:**

The aim of the study was to compare two Descemet membrane endothelial keratoplasty (DMEK) graft preparation techniques, the liquid bubble technique and the Melles technique.

**Methods:**

In this prospective study, a total of 20 corneas from 10 donors were included. For each donor, one cornea was prepared using the liquid bubble technique, and the contralateral cornea using the Melles technique. Outcome measures included endothelial cell count, the occurrence of tears and defects in the graft, preparation time, and histological findings.

**Results:**

Mean endothelial cell density (ECD) before preparation was 2330 ± 210 cells/mm² in the liquid bubble group (LB) and 2380 ± 157 cells/mm² in the Melles group (M). Mean ECD after preparation was 2170 ± 264 cells/mm² (LB) and 2287 ± 237 cells/mm² (M). This corresponds to an average cell loss during the preparation process of 160 ± 316,1 cells/mm² (6.87%, LB), and of 93 ± 147,1 cells/mm² (3,92%, M), which was not statistically significantly different (*p* = 0.05). Preparation time was statistically significantly different (*p* = 0.002) with a mean of 5.74 ± 2.43 min (LB) and 23.60 ± 1.81 min (M). In the liquid bubble group, collagen fibrils on the Descemet membrane after preparation were observed in 4 cases (40%), whereas in the Melles group, this was seen in only one case (10%).

**Conclusion:**

DMEK grafts prepared via the liquid bubble technique and via the Melles technique exhibit comparable ECD, graft quality, and surgical suitability. The liquid bubble technique offers a significant advantage in preparation time for donors without previous cataract surgery.

## Key Message

*****What is known***:**

- Descemet membrane grafts can be prepared using different techniques.
- Techniques are based on the use of forceps, fluids and air.
- The forceps technique is among the most frequently used methods.

*****What is new***:**

- Liquid bubble technique showed comparable quality outcomes to the forceps technique in a real-life-setting, confirming non-inferiority.
- Liquid bubble technique demonstrated significantly reduced preparation time.
- Collagen fibrils on the Descemet membrane were observed more frequently using the liquid bubble technique than with the Melles technique.

## Introduction

Descemet membrane endothelial keratoplasty (DMEK) has become the most commonly performed type of corneal transplantation for Fuchs Endothelial Corneal Dystrophy, due to its excellent visual outcomes and low rejection rates [1, 2]. DMEK surgery is expected to remain the standard for the foreseeable future, as pharmacological, regenerative, and genetic approaches are still unvalidated and far from clinical use [3].

A crucial step in DMEK is the precise preparation of the graft, which involves the separation of the Descemet membrane (DM) and its attached endothelial cell layer from the underlying corneal stroma [4]. This can lead to tears and mechanical stress, the quality and efficiency of this preparation significantly influence both surgical outcomes and endothelial cell viability [5].

Several techniques for preparing DMEK grafts have been developed. The traditional Melles technique relies on manual dissection using forceps [6]. The Submerged Cornea Using Backgrounds Away (SCUBA)-technique involves graft preparation using forceps while the corneal tissue is submerged in fluid [7]. The liquid bubble technique, utilizes fluid injection to create a cleavage plane between Descemet membrane and stroma [8]. A related, but older approach, the Teichmann technique, uses air or gas to generate a bubble, although this has been associated with more erratic results due to air entering the stroma [9, 10]. However, these methods can be broadly classified into two main approaches: manual DM peeling using forceps like the Melles techniques, and fluid- or air-assisted separation of the DM like the liquid bubble technique [4].

By direct comparison of the liquid bubble technique with the Melles technique in terms of preparation time, graft quality and endothelial cell count, this study aimed to identify the more efficient and cell-preserving technique for DMEK graft preparation.

## Methods

### Study design

This prospective experimental study was conducted in accordance with the Declaration of Helsinki, with approval from the ethics committee of the Medical Faculty of the University of Tübingen (project no. 183/2020BO2). Consent from the next of kin for using the tissues for research was obtained.

### Donor tissue

A total of 20 human donor corneas were included in the study. Corneal tissues were obtained from donors that were deemed unsuitable for transplantation due to contraindications. These included infectious diseases such as hepatitis, COVID-19-infection, or leukemia as a comorbidity. Patients with prior cataract surgery or with diabetes were not excluded. All donor corneas were preserved as corneoscleral discs in organ culture (culture medium I, fetal bovine serum, Pan-Biotech, Aidenbach, Germany) in culture flasks with possible gas exchange, at 5% CO₂, 95% humidity and at a storage temperature of 37 °C. All donor corneas met the quality criteria of a minimum endothelial cell density (ECD) of 2.000 cells/mm² prior to preparation. Before the preparation, the grafts were not transferred into a dextran-containing medium. After preparation, the transplant was stored in the same medium under identical conditions (37 °C, 5% CO₂, 95% humidity), but in 12-well plates with lids, which also allowed gas exchange.

### Preparation techniques

Corneas from each patient were randomly assigned so that one eye of each patient was allocated to the Melles technique preparation group and the contralateral eye to the Liquid Bubble Technique preparation group, ensuring paired comparison within subjects.

In the Liquid Bubble technique Group (*n* = 10), the grafts were prepared using the technique described by Szurman [5]. Briefly, a sharp dissection is performed at the iris base using a paracentesis knife to reach the Schlemm’s canal. Subsequently, further advancement is carried out with a blunt spatula centrally to Schwalbe’s line. A controlled volume of dye (VisionBlue, DORC Dutch Ophthalmic Research Center B.V., Zuidland, The Netherlands) was injected between the Descemet membrane and the stroma using a Sautter cannula to create a separation bubble; afterwards, the dye is released and the graft area is then trephined using an 8 mm trephine.

In the Melles technique group (*n* = 10) the grafts were prepared using the technique described by Melles [6]. Briefly, the trabecular meshwork is first selectively stained, followed by a sharp incision and blunt dissection toward the center using a specialized hockey knife. Due to strong adhesions between Descemet’s membrane and the underlying stroma near Schwalbe’s line, mechanical scraping is required in this region before the membrane can be easily peeled centrally with forceps, then repositioned, the sub-Descemet fluid gently dried, and the graft area is then trephined using an 8 mm trephine.

All procedures were performed by the same experienced surgeon under standardized conditions.

### Outcome Measures

#### Endothelial cell density

The ECD was evaluated after enucleation, within 48 h before and within 48 h after DM dissection. To improve visualization of the cells, the lamellae were placed in hypotonic balanced salt solution (BSS). Endothelial cell counts were performed using phase-contrast microscopy (Eclipse TS 100, Nikon, Amstelveen, The Netherlands) at 100× magnification and ImageJ (National Institutes of Health, Bethesda, MD). The fixed-frame method was used, two areas of 0.01 mm² each (100 × 100 μm field) were counted, for cell counting of the transplant after preparation, three areas were counted due to challenging counting conditions. The counting was performed by a single investigator with several years of experience and validation by a second experienced examiner.

#### Preparation Process

The preparation time was defined as the time (in minutes) from the start of manipulation to the complete detachment of the graft. Tears in the tissue during preparation were assessed. Additionally, the required number of sub-Descemet fluid injections (liquid bubbling) and the need for manual re-preparation were recorded [5, 9]. It was assessed whether membranes adhered to the graft.

#### Graft quality

The degree of tissue scrolling in the culture medium was evaluated, ranging up to the formation of a double roll. It was assessed whether any tears in the transplant were present after trephination.

#### Live/Dead Staining

Cell viability was assessed using a live/dead cell staining. The lamellae were incubated at room temperature for 30 minutes with Calcein Acetomethoxy (5 µM, Enzo Life Sciences, Lausen, Switzerland) and Propidium Iodide (15 µM, Sigma-Aldrich, St. Louis, Missouri, USA) in phosphate-buffered saline (PBS). They were then washed with PBS and fixed with 4% paraformaldehyde (PFA) for 10 minutes. After an additional PBS wash, the lamellae were incubated with DAPI (4’,6-diamidino-2-phenylindole, Sigma-Aldrich, St. Louis, Missouri, USA) for 5 min. Following staining, the lamellae were carefully mounted on a microscope slide in Fluorsafe and covered with a coverslip. Analysis was performed using fluorescence microscopy at 100× magnification (Axio Observer, Carl Zeiss Microscopy GmbH, Oberkochen, Germany) by two investigators. The aim was to verify that the counted cells were viable cells.

#### Histological examination

The prepared tissue was placed in 4% buffered neutral formaldehyde solution; subsequently, HE (Hematoxylin and Eosin) staining and PAS (Periodic Acid–Schiff) staining of the tissue were performed. The grafts were assessed for the presence of stroma/collagen lamellae, Descemet’s membrane splitting, the presence of guttae, and endothelial condition. The images were taken with a digital microscope (VHX-5000, Keyence Corporation, Osaka, Japan).

### Data analysis

Wilcoxon matched-pairs signed rank test was used to calculate potential differences between both preparation techniques. Spearman’s rank correlation test was used to determine the correlation between the occurrence of tears, age of donors and preparation time. A p-value of less than 0.05 was considered statistically significant. Data analysis was performed with SPSS (IBM SPSS Statistics for Windows, Version 28.0., released 2021, IBM Corp, Armonk, New York, USA).

## Results

### Corneal donor characteristics

Mean age of the donor corneas was 65.80 years (range 40–86 years). Nine donors were male (90%), one donor was female (10%) (Table 1). One donor had previously undergone bilateral cataract surgery. Two donors had diabetes mellitus. The causes of death included heart failure, carcinoma, leukemia, septic shock, pneumonia, and cerebral hemorrhage. The time between death and retrieval of the cornea was within 24 h. Mean graft storage time in culture medium was 23.60 days (± 8.79).

**Table 1:** The individual corneas with details of the preparation technique, cell count at the start of cultivation, before preparation, after preparation, preparation-specific observations, and histology

Cornea no.	Age of donor	Post-cataract surgery	Diabetes mellitus	Preparation technique	Graft storage time [days]	Cells at culture initiation [cells/mm²]	Cells before preparation [cells/mm²]	Cell loss while in culture [%]	Cells after preparation [cells/mm²]	Cell loss during preparation [%]	Time of preparation [min]	No. of bubblings	Manual repreparation	Adherent membranes	No. of tears	Defects in transplant	Scrolling of transplant	Stroma/collagen lamellae	Descemet-splitting	Endothelium	Guttae
1a	60	no	no	LB	27	1950	2000	3	1900	−5	3.53	2	0	0	0	0	double roll	no	no	good	no
1b	60	no	no	M	27	2100	2000	−5	1733	−13	13.38	n/a	0	0	3	0	double roll	no	no	good	no
2a	86	no	yes	LB	15	2450	2250	−8	2300	−2	7.52	0	1	0	1	2	none	yes	no	good	no
2b	86	no	yes	M	15	2400	2350	−2	2300	−2	11.88	n/a	0	0	3	2	poor	no	no	good	no
3a	63	no	no	LB	26	3050	2400	−21	2267	−6	3.50	2	0	0	0	0	double roll	no	no	good	no
3b	63	no	no	M	26	3250	2400	−26	2467	−3	15.15	n/a	0	0	1	0	double roll	no	no	good	no
4a	73	no	no	LB	22	2800	2350	−16	2700	−15	2.87	1	0	0	0	0	double roll	no	no	good	no
4b	73	no	no	M	22	2700	2450	−9	2600	−6	15.28	n/a	0	0	2	2	double roll	no	no	good	no
5a	63	no	no	LB	31	2750	2150	−22	2033	−5	7.02	2	0	0	0	0	double roll	yes	no	good	no
5b	63	no	no	M	31	2600	2300	−12	2100	−9	14.00	n/a	0	0	2	2	fair	yes	no	good	no
6a	68	no	no	LB	13	2750	2650	−4	2000	−25	5.07	1	0	0	0	0	double roll	yes	no	good	no
6b	68	no	no	M	13	2750	2550	−7	2367	−7	18.13	n/a	0	0	0	0	double roll	no	no	good	no
7a	78	yes	yes	LB	31	3200	2600	−19	1933	−26	11.07	2	1	0	2	0	double roll	yes	no	good	no
7b	78	yes	yes	M	31	2650	2550	−4	2233	−12	15.12	n/a	0	0	5	2	poor	no	no	good	no
8a	40	no	no	LB	33	2900	2350	−19	2467	5	6.00	3	0	0	0	0	double roll	no	no	good	no
8b	40	no	no	M	33	2900	2400	−17	2333	−3	15.03	n/a	0	0	0	0	double roll	no	no	good	no
9a	61	no	no	LB	31	2300	2100	−9	1933	−8	6.05	3	0	0	0	0	fair	no	no	good	no
9b	61	no	no	M	31	2500	2350	−6	2300	−2	12.02	n/a	0	0	0	0	poor	no	no	good	no
10a	66	no	no	LB	7	2650	2450	−8	2167	−12	4.75	2	0	0	0	0	double roll	no	no	good	no
10b	66	no	no	M	7	2750	2450	−11	2433	−1	13.95	n/a	0	0	1	0	double roll	no	no	good	no

*LB* liquid bubble preparation technique, *M *Melles preparation technique, *n/a *not applicable

### Preparation process

**Fig. 1:**
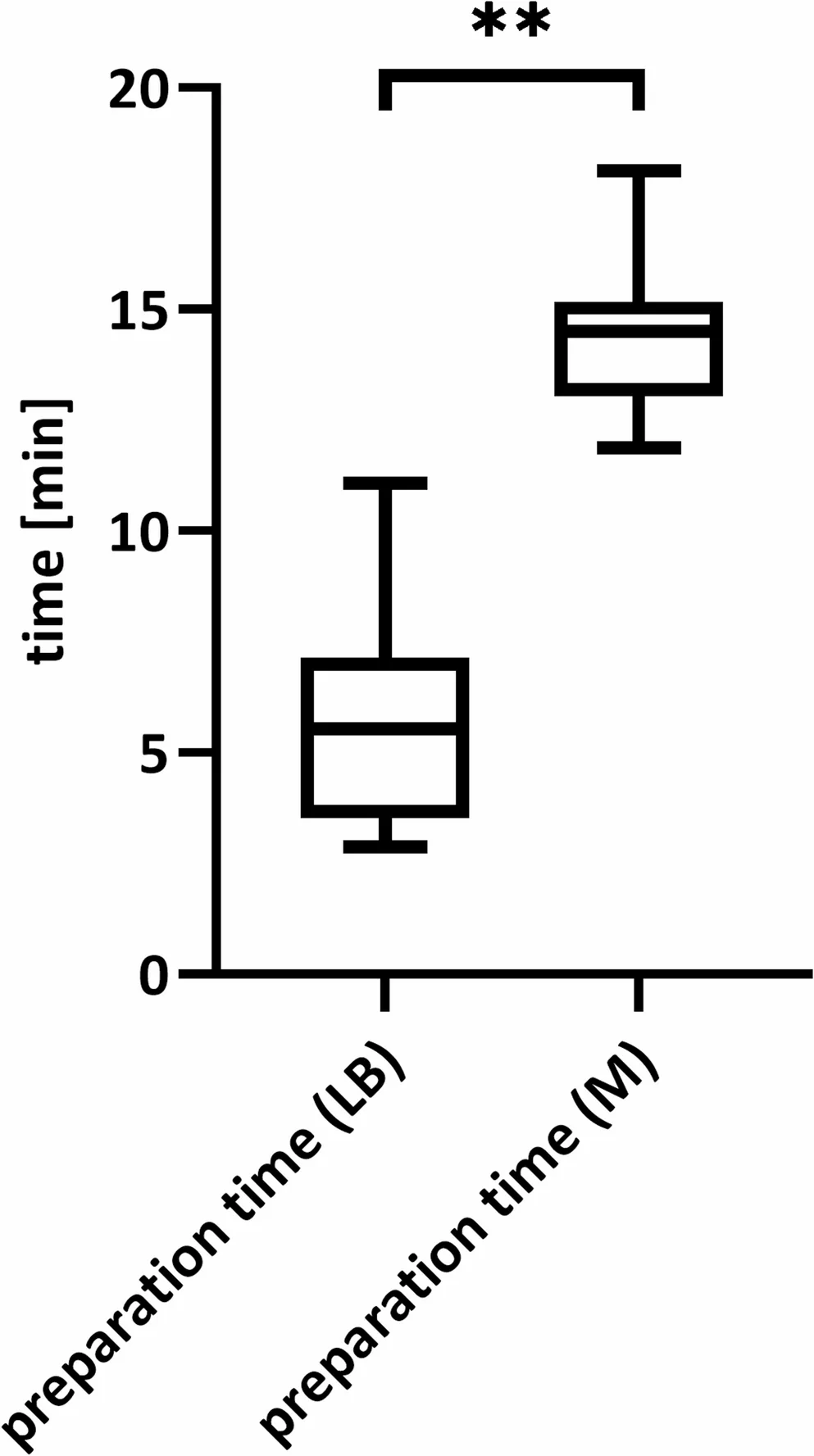
Preparation time for liquid bubble technique (LB) and Melles technique (M); LB was statistically significantly faster (*p* = 0.0020; *n* = 20)

Mean preparation time in liquid bubble technique was 5.74 ± 2.43 min (median: 5.54 min), in Melles technique 14.39 ± 1.81 min (median: 14.52 min) (Fig. 1). This difference in preparation time was statistically significant (*p* = 0.0020, *n* = 20, Wilcoxon-test). After exclusion of donor 7, the only tissue donor with a history of cataract surgery and a markedly longer preparation time for the liquid bubble technique compared to the others donors, the difference in preparation time was more pronounced, with 5.15 ± 1.63 min for the liquid bubble technique (median: 5.07 min) and 14.31 ± 1.90 min for the Melles technique (median: 14.00 min). Accordingly, this difference was also statistically significant (*p* = 0.0039, *n* = 18, Wilcoxon-test). The average number of bubblings in liquid bubble technique was 1.80. Manual re-preparation after liquid bubble preparation was necessary in two cases (20%), one of them was an eye with a history of cataract surgery. Adherent membranes were not seen in any case.

### Graft quality

In the liquid bubble group, tears were observed in only two cases (20%), with one case showing a single peripheral tear and the other case showing two central tears. In Melles’ group, tears occurred in seven cases (70%), with up to five tears per case, most of which were in the periphery. This difference is statistically significant (*p* = 0.0156, Wilcoxon-test). There was a statistically significant positive correlation between the age of the donors and the occurrence of tears (*p* = 0.0348, Spearman *r* = 0.4738) as well as between the age of the donors and the number of tears (*p* = 0.0392, Spearman *r* = 0.4643). There was also a statistically significant positive correlation between the preparation time and the occurrence of tears (*p* = 0.0122, Spearman *r* = 0.5490) as well as between the preparation time and the number of tears (*p* = 0.0165, Spearman *r* = 0.5289). The patient with a previous cataract surgery revealed tears, as did the two patients with diabetes mellitus.

In the liquid bubble group, only one case exhibited two defects after trephination. In the Melles group four patients showed defects after trephination, with two defects per patient. This difference is not statistically significant (*p* = 0.25, Wilcoxon-test).

The degree of tissue scrolling in the culture medium was evaluated in the liquid bubble group as double roll in 8 cases, in one case scrolling of the transplant was evaluated as fair, one case revealed no scrolling of the transplant. In the Melles group the tissue scrolling was evaluated as double roll in six patients, in one as fair and in three as poor. The patients with diabetes mellitus showed no double-roll formation after preparation in 3 out of 4 eyes and thus accounted for 50% of the cases without double-roll formation.

### Endothelial cell density

**Fig. 2:**
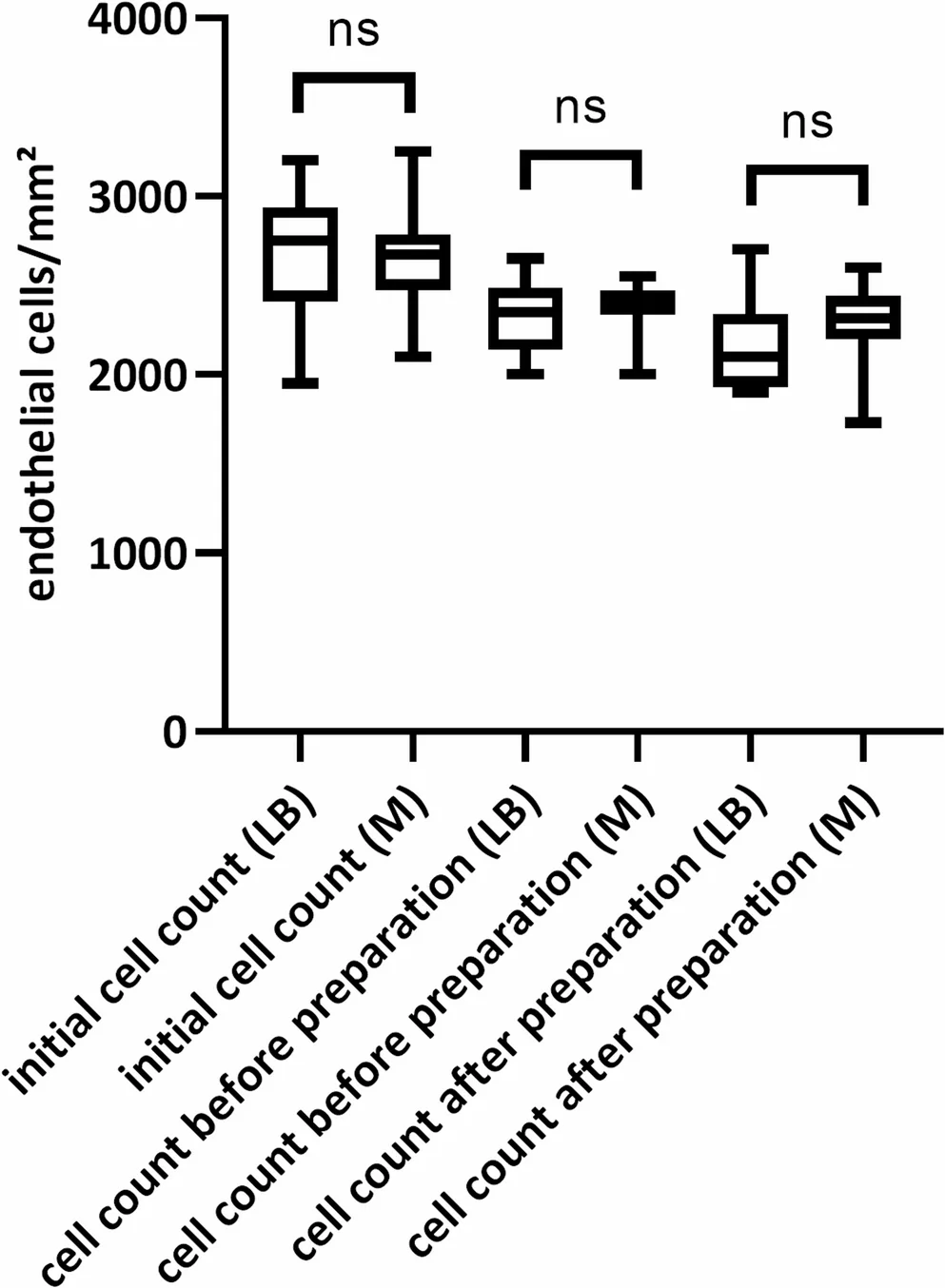
Endothelial cell density at start of organ culture, before preparation, and after preparation with liquid bubble (LB) or Melles (M). Cell density declined continuously, with a greater loss during cultivation than during preparation. No significant differences were found between both techniques (start: *p* = 0.8281; before preparation: *p* = 0.2031; after preparation: *p* = 0.1250)

The initial ECD at the time of culture initiation was 2680 ± 367 cells/mm² in the liquid bubble group and 2660 ± 304 cells/mm² in the Melles group (Fig. 2). There was no statistically significant difference between the two groups (*p* = 0.83, Wilcoxon-test).

The mean ECD before preparation was 2330 ± 210 cells/mm² in the liquid bubble group and 2380 ± 157 cells/mm² in the Melles group. There was no statistically significant difference in ECD before preparation between the two groups (*p* = 0.20, Wilcoxon-test). This corresponds to a mean endothelial cell loss between culture initiation and the start of preparation of 350 ± 248 cells/mm² (13.06%) in the liquid bubble group and of 280 ± 239 cells/mm² (10.53%) in the Melles group. The cell loss in each group was statistically significant (liquid bubble group: *p* = 0.0039; Melles group: *p* = 0.0020), however, without any statistically significant difference between the groups (*p* = 0.4824, Wilcoxon-test).

The mean ECD after preparation was 2170 ± 264 cells/mm² in the liquid bubble group and 2287 ± 237 cells/mm² in the Melles group. This corresponds to an average cell loss during the preparation process of 160 ± 316 cells/mm² (6.87%) in the liquid bubble group, and of 93 ± 147 cells/mm² (3,91%) in the Melles group. However, the endothelial cell loss during the preparation process was not statistically significant (liquid bubble group: *p* = 0.1387; Melles group: *p* = 0.0918), there was neither a statistically significant difference in cell loss between both techniques (*p* = 0.3887, Wilcoxon-test).

### Live/Dead staining

**Fig. 3:**
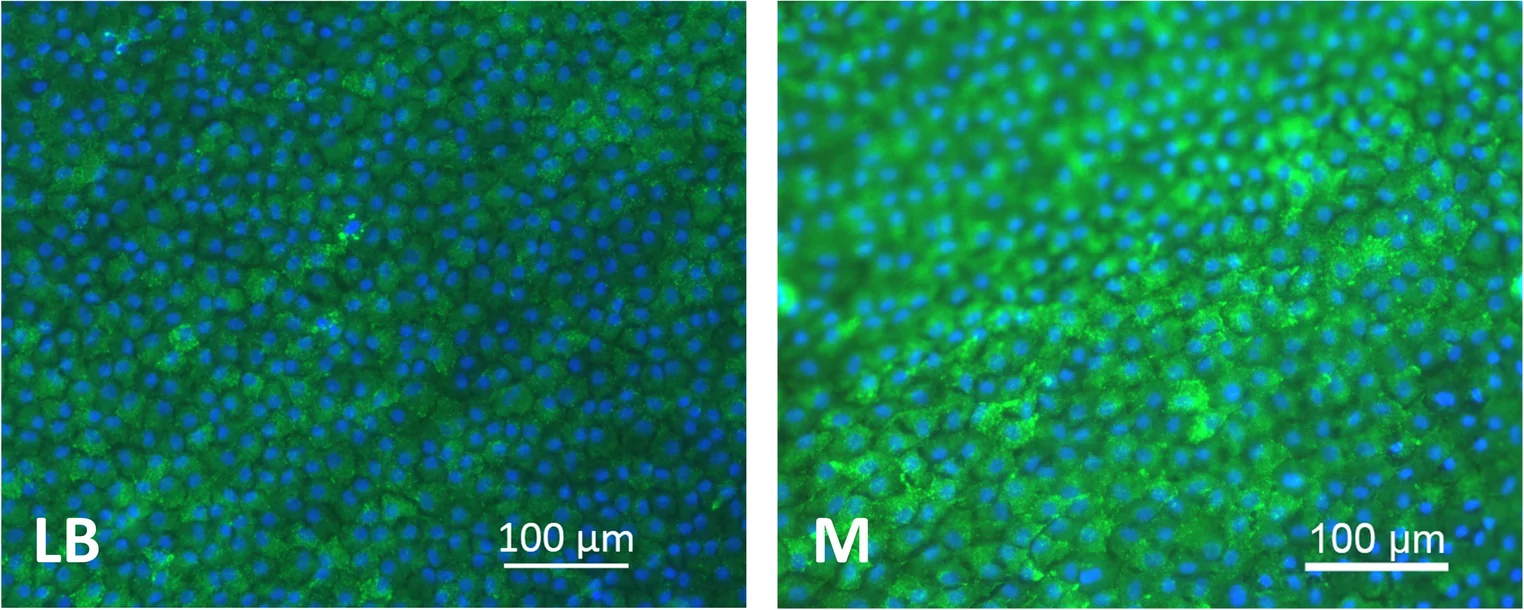
The Live/Dead staining of a cornea prepared using the liquid bubble technique (LB, patient 4a) and a cornea prepared using the Melles technique (M, patient 4b). Note that almost exclusively living cells are present

The Live/Dead staining revealed no or only a very small proportion of dead cells (Fig. 3). Accordingly, the counted cells were viable cells and therefore potentially suitable for transplantation.

### Histological examination

**Fig. 4:**
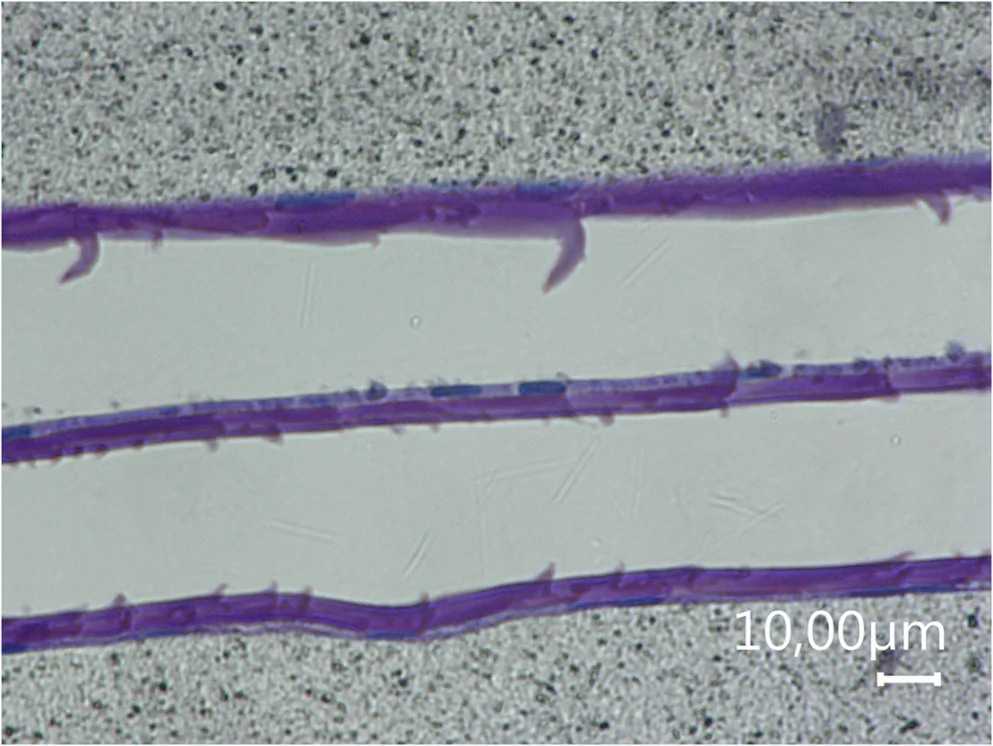
Note the collagen fibrils on the Descemet membrane of the graft (Periodic Acid–Schiff staining, patient 2a). Comparable attachments of collagen fibrils were observed after preparation in 4 cases when using liquid bubble technique, whereas only in one case when using Melles technique

In the liquid bubble group, collagen fibrils on the Descemet membrane were observed in 4 cases (40%) after preparation (Fig. 4), whereas in the Melles group, this was seen in only one case (10%), that is without statistically significant difference (*p* = 0.25, *n* = 20, Wilcoxon-test). No Descemet membrane splitting was observed in any of the tissues. Histologically, the endothelium was in good condition, and guttae were not detected.

## Discussion

The preparation of DMEK grafts is a crucial step influencing surgical success and patient outcomes [11]. Several techniques for DMEK graft preparation have been described in the literature [4, 11, 12]. Determining the most suitable preparation technique, especially regarding graft quality and required time, has become an area of ongoing debate, as each technique offers distinct advantages and potential drawbacks.

### Preparation process

The preparation time differed significantly between the two techniques, with 5.54 min for the liquid bubble technique compared to 14.52 min for the Melles technique. The markedly shorter duration associated with the liquid bubble approach suggests a potentially more efficient preparation process and is in agreement with previous studies, that report 3.23 min and 7.90 min for hydrodissection [5, 13] compared to 11.00 min and 9.43 min for the Melles technique [12, 14]. From a practical standpoint, the time-saving advantage of the liquid bubble technique proves particularly decisive in surgical settings where the surgeon prepares the graft, improving workflow efficiency and reducing tissue handling time, which might indirectly benefit endothelial survival.

If the liquid bubble technique fails at the initial injection site for example due to a small tear or adherent membranes, the procedure can be reattempted from a different location. Secondary manual preparation may be applied as a rescue technique, thereby minimizing the risk of terminating the procedure due to insufficient progress. Manual re-preparation after liquid bubble preparation was required in two cases (20%), which is in line with comparable studies reporting a re-preparation rate of 25% [13], however, higher than the 5% reported in the study that first described the method [5]. One of the main reasons is previous cataract surgeries, as prior cataract incisions prevent bubble formation, considerably complicating preparation using the liquid bubble technique [12]. Consistent with this, in the present study, manual re-preparation was necessary in one of the two eyes with a history of cataract surgery.

As a result of these different rescue strategies in cases of small tears or inadequate progress (bubbling from different sides, manual re-preparation), in the present study, all tissues could be successfully utilized, and no tissue loss occurred, consistent with previous reports [13]. Likewise, no tissue loss was observed with the Melles technique in this study, although other reports describe higher loss rates of 7% [15] or up to 10% [16] due to diffuse adhesions and radial tears extending into the central zone.

### Graft quality

In the liquid bubble technique group, tears were observed in only two cases (20%), whereas in the Melles technique group, tears occurred in seven cases (70%). The small proportion of grafts with tears in the liquid bubble technique group is consistent with previous reports, some of which observed no tears at all when applying the hydrodissection technique [13]. A significantly higher rate of tears was observed during preparation with the Melles technique, in line with previous studies reporting tears in approximately 15% of grafts [16], also manual preparation under water showed substantially higher tear rates of 25% [17].

The number of tears per graft was no more than two with the liquid bubble technique, whereas the Melles technique resulted in up to five per tissue, which is consistent with previous studies reporting a mean of 3.1 radial tears per graft with the Melles technique [16].

However, most of the tears were located in the peripheral area in line with earlier findings [5], so that, after trephination, no statistically significant differences in defect frequency were observed between the two techniques.

The reason for the higher rate of peripheral tears with the Melles technique compared to the liquid bubble technique can only be hypothesized. One possible explanation is that the liquid bubble technique separates the Descemet membrane more evenly from the posterior stromal lamella within the natural interfacial matrix, creating a more homogeneous layer [18]. However, histological analysis of our grafts revealed slightly more collagen attachments with the liquid-bubble technique. Therefore, the difference is more likely related to mechanical stress during the preparation process. In the manual technique, localized shear forces are applied by the forceps, whereas the liquid bubble method uses hydraulic forces to gently lift the tissue from below. This might produce lower local traction and reduce the risk of microtears caused by small residual stromal fibers, which may enlarge under tension.

Moreover, manual preparation is highly surgeon dependent. Experience with the method, forceps handling, and the angle of traction all play a critical role. In contrast, the liquid bubble technique is more standardized, largely independent of the operator [5].

In addition, previous corneal procedures might be relevant. Prior cataract incisions increase the risk of tearing [12]. In line with this, In the present study, the patient with a history of cataract surgery showed the highest number of tears in the graft, particularly when preparing with the Melles technique. Tear formation might also be influenced by donor comorbidities like diabetes [19] and the age of the donor. Consistent with this, in the present study, all patients with diabetes mellitus exhibited tears during preparation, both with the liquid bubble technique and the Melles technique.

Donor age is supposed to play no significant role, with reports of equal rates of graft tears across donor ages when using the forceps-based preparation technique [20]. In contrast, our study showed a statistically significant correlation between the age of the donors and the occurrence of tears, particularly with the liquid bubble technique, which produced tears only in the two oldest patients. One possible explanation for this could be that the corneal tissue reveals a reduced capacity for deformation, as it becomes more resistant with increasing age [21].

However, the donor-specific conditions were controlled in this study, as paired corneas from the same donor were used for comparison, also a strong correlation between the two eyes from the same donor regarding the preparation process has been reported [20].

A pronounced double-roll configuration was observed predominantly in the liquid bubble group (80%), whereas in the Melles group the double-roll pattern was less frequent (60%) and moderate or even absent scrolling was recorded more often. The causes of these differences in scrolling behavior can only be hypothesized.

The tendency of the Descemet membrane to roll appears to be less influenced by residual posterior stromal tissue, as the liquid bubble technique showed more stromal attachments yet demonstrated better scrolling.

Instead, changes in the mechanical tension of the Descemet membrane through local traction forces during the preparation process in Melles technique may play a key role. In contrast, the liquid bubble technique releases the lamella largely without pressure, helping to preserve its natural elastic tension.

Other contributing factors may involve endothelial surface properties, which can be affected by staining, hydration or exposure to air, and may alter the surface tension. Differences in staining duration between both techniques could therefore modulate scrolling behavior. In addition, the considerably longer preparation time required for the Melles technique may cause localized dehydration, potentially resulting in irregular scrolling patterns. Comorbidities of the donors could also be relevant here, as in the present study, patients with diabetes mellitus showed no double-roll formation after preparation in 3 out of 4 eyes and thus accounted for 50% of the cases without double-roll formation.

### Endothelial cell density

ECD is particularly relevant for graft survival [22]. The average cell loss during preparation was higher in the liquid bubble group compared to the Melles group, but without statistical significance. The liquid bubble technique avoids direct contact between endothelial cells and surgical instruments and allows selective staining restricted to the stromal side, thereby protecting the endothelium from dye exposure [5]. A possible explanation for observed cell damage, however, may be the mechanical stress caused by the pressure of the injected fluid, stretching the tissue and potentially causing cell damage [23]. It is conceivable that the Melles technique might result in even lower cell loss if the endothelium is kept continuously covered with fluid during preparation, thereby preventing desiccation [24].

The endothelial cell loss during cultivation exceeded that observed during preparation, suggesting that the preparation technique was less decisive for cell count preservation - particularly in corneas stored for extended periods, in which considerable endothelial damage had already occurred prior to preparation. In line with this, the mean cultivation time in the present study was relatively long, averaging 23.6 days.

### Histological examination

Overall, the Melles technique, with collagen fibrils on the Descemet membrane in one case (10%), achieved better separation of the Descement membrane from the stromal tissue than the liquid bubble technique with collagen fibrils on the Descemet membrane in four cases (40%), however, without statistical significance. These remnants of collagen fibrils are potentially relevant for graft detachment or transplant failure [25, 26].

The presence of a small amount of collagen fibrils after applying the liquid bubble technique has been reported before [9], others report no stromal residues [18]. A possible explanation for the difference may lie in the preparation process of the liquid bubble technique. During liquid bubble preparation, the injection of liquid is not always delivered directly beneath the Descemet membrane, but sometimes into the stroma. In such cases, the liquid still generates sufficient force to cleave stromal tissue and reach the stroma–Descemet membrane (DM) interface, forming the desired bubble [9, 12]. However, as the liquid dissects through the stroma, residual collagen fibrils may remain.

### Strengths and limitations of the study

The key strength of the study is that corneas from the same donor could be compared, prepared on the same day by the same surgeon using both techniques under investigation. The study was conducted in a real-world clinical setting and apart from the individual contraindications of the donors, the grafts met all the requirements for transplantation.

A limitation of the study is the comparatively small number of grafts included. However, this is within the range of comparable studies, and achieving a higher number is difficult given the limited availability of donors. Nine out of ten donors had no prior cataract surgery; therefore, the results can primarily be attributed to donors without previous cataract surgery, particularly with regard to the reduced preparation time in liquid bubble technique. It should also be noted that in our routine clinical practice the liquid bubble technique is predominantly employed, resulting in considerably greater experience and familiarity of the surgeon with this approach. Furthermore, all preparations were performed by a single experienced surgeon, which may limit generalizability. Comorbidities that led to the exclusion of the graft from transplantation may affect the graft quality.

## Conclusion

Graft quality, endothelial cell density (ECD), and overall suitability for DMEK surgery were comparable between the liquid bubble technique and the Melles technique, indicating that both approaches yield clinically usable grafts. The liquid bubble technique offers a significant advantage in preparation time for donors without previous cataract surgery, highlighting its potential to increase efficiency in surgical and eye bank settings that allow the exclusive selection of non-cataract donors.

## Data Availability

The data that support the findings of this study are available on request from the corresponding author. The data are not publicly available due to privacy or ethical restrictions.
